# The use of low-cost Android tablets to train community health workers in Mukono, Uganda, in the recognition, treatment and prevention of pneumonia in children under five: a pilot randomised controlled trial

**DOI:** 10.1186/s12960-018-0315-7

**Published:** 2018-09-19

**Authors:** James O’Donovan, Kenneth Kabali, Celia Taylor, Margarita Chukhina, Jacqueline C. Kading, Jonathan Fuld, Edward O’Neil

**Affiliations:** 1 Department of Medicine, Addenbrookes Hospital, Cambridge, UK; 2 Department of Education, University of Oxford, Oxford, UK; 3 Division of Research, Omni Med, Mukono, Uganda; 4 Division of Health Sciences, Warwick University Medical School, Coventry, UK; 5 School of Medicine, Washington University School of Medicine in St. Louis, St Louis, USA; 6 Department of Emergency Medicine, Steward St Elizabeth’s Medical Center, Boston, USA; 7 Tufts University School of Medicine, Tufts University, Boston, USA

**Keywords:** Community health worker (CHW), Uganda, Pneumonia, mHealth, Mobile technologies, Integrated Community Case Management (iCCM)

## Abstract

**Background:**

Since 2012, The World Health Organization and UNICEF have advocated for community health workers (CHWs) to be trained in Integrated Community Case Management (iCCM) of common childhood illnesses, such as pneumonia. Despite the effectiveness of iCCM, CHWs face many barriers to accessing training. This pilot study compares traditional training with using locally made videos loaded onto low-cost Android tablets to train CHWs on the pneumonia component of iCCM.

**Methods:**

We conducted a pilot randomised controlled trial with CHWs in the Mukono District of Uganda. The unit of randomisation was the sub-county level, and the unit of analysis was at the level of the individual CHW. Eligible CHWs had completed basic iCCM training but had not received any refresher training on the pneumonia component of iCCM in the preceding 2 years. CHWs in the control group received training in the recognition, treatment, and prevention of pneumonia as it is currently delivered, through a 1-day, in-person workshop. CHWs allocated to the intervention group received training via locally made educational videos hosted on low-cost Android tablets. The primary outcome was change in knowledge acquisition, assessed through a multiple choice questionnaire before and after training, and a post-training clinical assessment. The secondary outcome was a qualitative evaluation of CHW experiences of using the tablet platform.

**Results:**

In the study, 129 CHWs were enrolled, 66 and 63 in the control and intervention groups respectively. CHWs in both groups demonstrated an improvement in multiple choice question test scores before and after training; however, there was no statistically significant difference in the improvement between groups (*t* = 1.15, *p* = 0.254). There was a statistically significant positive correlation (Pearson’s *r* = 0.26, *p* = 0.03) linking years of education to improvement in test scores in the control group, which was not present in the intervention group. The majority of CHWs expressed satisfaction with the use of tablets as a training tool; however, some reported technical issues (*n* = 9).

**Conclusion:**

Tablet-based training is comparable to traditional training in terms of knowledge acquisition. It also proved to be feasible and a satisfactory means of delivering training to CHWs. Further research is required to understand the impacts of scaling such an intervention.

**Trial registration:**

Registered on 23/11/2016 at clinicaltrials.gov (NCT02971449).

**Electronic supplementary material:**

The online version of this article (10.1186/s12960-018-0315-7) contains supplementary material, which is available to authorized users.

## Background

The World Health Organization (WHO) has forecast a global shortage of 18 million health workers by 2030 [1]. One solution proposed by the WHO to address this gap has been to advocate for the recruitment, training and deployment of community health workers (CHWs) in low- and middle-income countries (LMICs) [2].

CHWs are lay people working within their own community in a health promotion, prevention and delivery role [3]. In 2002, Uganda began implementing a national CHW program known as village health teams (VHTs) [3]. In the VHT model, CHWs are locally elected and are given the responsibility of caring for between 25 and 30 households. Their primary responsibilities include sharing preventive health measures, referring sick patients to local health centres, and collecting health data for the Ministry of Health (MoH) [3]. In 2012, the MoH expanded the role of CHWs to include treatment of the three leading killers of children under age five in Uganda: diarrhoea, malaria and pneumonia. This program, called Integrated Community Case Management (iCCM), has been endorsed by WHO and UNICEF as an equity-focused strategy seeking the prevention and early case detection of diarrhoea, malaria and pneumonia in the community [4, 5]. The traditional iCCM approach in Uganda involves CHWs attending a 5-day training course in one location, traditionally taught didactically by a CHW supervisor, followed by a 1-day refresher course every 6 months [6].

Despite the need for refresher training, many challenges surround its provision, including a shortage of trained personnel to deliver training, lack of attention from government-level policy makers, financial constraints and long gaps between initial and refresher training [7, 8]. Lack of refresher training is one factor that has been identified as contributing to the failure of CHW programs over time [9, 10]. One study assessing challenges faced by CHWs in Northern Uganda found that 18% had not received scheduled refresher training since recruitment, and those that had received it reported erratic external support from a variety of non-governmental organisations [7]. Best practice guidelines produced by the USAID Health Care Improvement Project to improve CHW program performance recommend that refresher training should be provided at least every 6 months to update CHWs on new skills, reinforce initial training, and ensure he or she is practicing skills learned [11].

Recent advances in technology offer a potential solution to the on-going challenges facing the provision of high-quality and accessible CHW refresher training. One example is the use of mobile health (mHealth) as a tool to deliver education to healthcare professionals [12]. mHealth can be defined as “interventions and programs designed to support medical and public health through the use of mobile technology” [13]. Although the term mHealth commonly refers to handheld mobile phone devices, it can also encompass other devices such as computer tablets [13]. Labrique et al. identified several core uses of mHealth interventions, one of which included mHealth as a means to provide continuous training and education [14, 15].

This study describes the use of low-cost Android tablets pre-loaded with locally made educational videos to train CHWs in rural Uganda in the recognition, treatment and prevention of pneumonia in accordance with iCCM guidelines. The hypothesis was that tablet-based training would be feasible, acceptable and comparable to traditional training in terms of knowledge acquisition regarding the cause, prevention and management of pneumonia amongst CHWs.

## Methods

### Setting and participants

The study took place in Mukono, a district in central Uganda, from 15 November 2016 to 15 December 2016 [16]. Mukono has a population of just under 600 000, with an estimated under-five population of 30% [17]. Regarding health indicators, Mukono has extremely poor neonatal mortality and malnutrition indicators [18]. In 2011, pneumonia was the leading cause of death amongst children under the age of five in the district, with a case fatality rate of 5 % [18]. This study was implemented by Omni Med, an NGO based in the United States and Uganda that has been training CHWs in Mukono District since 2009 and adapting the iCCM strategy with Ministry of Health approval since 2012 (http://www.omnimed.org/).

CHWs were recruited from three sub-counties in the district: Mpatta, Nakisunga and Mpunge. These areas were selected as locations since CHWs in all three sub-counties had received initial training from Omni Med staff but had not received any iCCM refresher training in the past 2 years. CHW supervisors in individual parishes were informed of the study by a formal letter delivered by the staff of Omni Med. CHW supervisors then had 14 days to notify the CHWs in their parish about the training.

In order to be eligible, CHWs had to have completed basic iCCM training but not have received any refresher training on the pneumonia component of iCCM in the preceding 2 years. There were no exclusion criteria based on age, gender, sex or tribe. Participants were assured of the right to accept or refuse to take part in the study without consequences. Those who agreed to take part signed an informed consent form, which was verbally translated into Luganda from English.

### Materials

The tablet used in the study was a $50 USD Amazon Kindle Fire 7 (8 GB). The tablets have a 7-in. screen and operate using an Android platform. Each tablet was housed in a “ruggedised” rubber carry case ($16 USD) to help prevent damage and issued with a solar charger ($15 USD), allowing individual CHWs the ability to charge the battery on their tablet device independent of having access to a source of mains electricity. This model was selected due to its relatively low cost, its expandable memory capacity of 256 GB which enabled us to store and host multiple high-quality instructional videos independent of internet access and high consumer reliability ratings.

Four instructional videos were created by Omni Med staff in July 2016 using up to date iCCM guidelines [19]. The videos were filmed in Mukono in the local dialect (*Luganda*) with English subtitles and featured local CHWs and a Ugandan physician (KK). The videos focused on (i) What is pneumonia? (ii) How to recognise pneumonia? (iii) How to treat pneumonia? and (iv) How to prevent pneumonia? [19]. The video tutorials were reviewed and approved by the Commissioner for Child Health at the MoH and two independent medical doctors for accuracy, before being preloaded onto the tablets.

### Study design

This was a pilot randomised controlled trial carried out over the period of 1-month. We adhered to the UNICEF “Principles for Innovation and Technology in Development” [20]. The unit of randomisation was the sub-county level, and the unit of analysis was at the level of the individual CHW. CHWs were randomly allocated to one of two groups (intervention or control) at a sub-county level. Sub-counties were assigned a number between one and three and a random number generator with the parameters of one to three was used. It was decided the first number to be drawn would be allocated as the control group and the next two as the intervention. Mpatta was assigned as the control arm and Mpunge and Nakisunga as the intervention arms. For further information regarding the control and intervention groups, please refer to the CONSORT diagram (see Additional file 1). Due to the nature of the intervention, CHWs could not be blinded to their group assignment.

Standard iCCM training covering all aspects of diarrhoea, malaria and pneumonia is traditionally carried out by trained instructors in didactic training modules over a period of 5 days. In this study, CHWs in the control group received didactic training delivered by Omni Med staff on the recognition, treatment and prevention of pneumonia in 1 day. Prior to commencing training, all CHWs completed a multiple choice questionnaire (MCQ) assessment. The training session was held in a classroom, and CHWs returned 6 days later to complete a post-training MCQ and practical assessment.

The CHWs allocated to the intervention group received training using the low-cost tablet devices with pre-loaded instructional videos. They attended a half-day workshop on a Monday, where an Omni Med staff member provided basic training on how to use the tablet and CHWs completed the same pre-training MCQ assessment as the control group. Upon returning the tablet after 5 days, CHWs completed the post-training MCQ and practical assessment, as well as a questionnaire regarding their experience of the program.

Both intervention and control groups received a reimbursement of approximately $5 USD for travel costs.

### Sample size calculation

We sought to detect an effect size of approximately 0.6 standard deviations (2 points on a test with a possible maximum score of 24 (8.3%), using a pooled standard deviation of changes in scores on a similar test used in a small, unpublished feasibility study and which was also marked out of 24 was 3.4). We sought to detect a larger effect size than the 0.4 standard deviations considered the minimum important difference for educational interventions in Hattie’s seminal work because of the resource limitations for any form of on-going training for CHWs and, indeed, for CHW programmes in general [21, 22].

The sample size calculation was generated using SAS software, Version 9.1.3 of the SAS System for Unix [23]. It was determined we would require 39 CHWs in each arm, to detect a difference of this magnitude, assuming a one-sided test with 80% power and a type I error rate of 5%. To allow for an estimated 25% dropout rate, we initially aimed to recruit 50 CHWs per arm in this pilot trial.

### Use of structured knowledge and clinical examination mark schemes

CHWs were assessed using a dual assessment. To assess theoretical knowledge before and after training, a MCQ with 24 questions was used (see Additional file 2). The questions in the MCQ test were developed using the iCCM syllabus and translated into Luganda. The questions on the post-training MCQ were the same as those asked in the pre-training MCQ to facilitate comparison of performance. CHWs were given 60 min to complete each MCQ.

To assess levels of practical and clinical knowledge acquisition after receiving training, we developed a clinical testing tool, utilising seven clinical cases (see Additional file 3). The cases consisted of a short clinical vignette, accompanied by a video, which contained a child under the age of five who either had normal or fast breathing. CHWs worked through the cases under the supervision of an independent assessor who was blinded to group assignment. Based on their assessment of the child’s respiratory rate and other clinical information contained within the cases, they answered between two and four questions per case regarding how they would manage the child. The answers given by the CHWs were recorded on a mark scheme by the independent assessor. CHWs were only assessed using the clinical assessment post-training.

MCQs and clinical case answer sheets were marked by two members of Omni Med staff who were blinded to group assignment using an answer key. This was double-checked for accuracy by an independent marker to verify the score. A score of 1 was given for each correct answer, and 0 for incorrect answers. There was no negative marking in either test. The responses for the theoretical knowledge MCQ tests and post training practical tests were uploaded to an Excel spread sheet and stored electronically for subsequent analysis.

### End user feedback

To elicit end user feedback from the CHWs regarding their views and experiences of tablet-based training, we administered a post-intervention questionnaire containing open-ended questions (see Additional file 4).

### Outcomes

The primary feasibility assessment outcome was knowledge acquisition and retention as defined by changes in MCQ scores (post-training score to pre-training score) and post-training clinical test scores for both the intervention and control groups. The secondary outcome measures assessed the experiences of CHWs using the tablet-based form of training.

### Statistical analysis

The primary analysis was per protocol. Statistical analysis was conducted using RStudio [24] and Stata v14 [25]. To compare the characteristics of the control and intervention groups, we used a chi-squared test with Yates’ continuity correction and two-sided two-sample *t* tests depending on the nature of the data.

Changes in MCQ scores were approximately normally distributed in both the control (*W* = 0.99, *p* = 0.74) and intervention groups (*W* = 0.99, *p* = 0.96) as determined by using a Shapiro-Wilk test. Differences in these outcomes between groups were analysed using one-sided independent samples *t* test. A one-sided test was used because we did not expect the intervention group to have inferior outcomes. Similarly, post-training clinical case test scores were also approximately normally distributed within the control (*W* = 0.97, *p* = 0.17) and intervention (*W* = 0.98, *p* = 0.35) groups, and thus the difference between groups was also analysed using a one-sided independent samples *t* test. To estimate the effect of participant demographics (age, gender, years as a CHW, number of children under the age of five, and years of education) on the change in MCQ scores, we undertook a univariate analysis for each demographic for each group using an appropriate statistical test. Any demographic variables statistically significant at *p* < 0.05 in either group were included in a single multiple linear regression, including group assignment and an interaction term between group assignment and the demographic variable to determine if the effect of the variable was consistent across groups. We also calculated the Pearson’s correlation between pre-training MCQ scores and MCQ change scores to determine if pre-training scores influenced propensity to improve. A *p* value of < 0.05 was considered statistically significant.

### Analysis of end-user feedback

All of the post-intervention questionnaires were collated, and frequency counts were used for closed questions, while open-ended question responses were analysed using thematic analysis. The analysis was performed through coding each individual sentence written by the CHW to look for meaningful patterns across the data. This was done by two authors (JOD and MC) who worked together to generate initial codes and subsequent themes. The phases of analysis were familiarisation with the data, generation of initial codes, searching for themes amongst codes, reviewing of themes, appropriately defining and naming themes, and writing up findings.

## Results

### Demographics

Two hundred thirty-eight CHWs were assessed for eligibility prior to the commencement of the trial using the inclusion and exclusion criteria. After this initial assessment, 75 CHWs declined to take part in the study because they could not be available in the final morning to take part in the post-intervention test (*n* = 54) or had prior commitments to attend to (*n* = 21). This left 163 CHWs across the three sub-counties who were eligible and willing to take part in the study. Eighty-six CHWs were in the Mpatta sub-county allocated to the control arm and 77 were in the Nakisunga and Mpunge sub-counties, which were allocated to the intervention arm. Unfortunately, 11 CHWs from the control arm and five from the intervention arm did not attend the initial assessment day after group allocation. Furthermore, nine CHWs were lost to follow-up in each arm since they became unavailable on the final morning to take part in the post-intervention test, leaving the total number of CHWs who completed the training as 66 and 63 in the control and intervention respectively (see Additional file 1).

Both groups were well matched in terms of demographics, with no significant differences between groups in any of the recorded variables (gender, age, number of years as a CHW, average number of children in the household and number of children in the household under-five) (see Additional file 5). One hundred percent of tablets issued over the course of the study were returned in full working order. No tablets were lost, stolen or misplaced.

### Multiple choice questionnaire scores

Independent samples *t* test revealed no significant differences between the control and intervention groups’ pre- or post-training MCQ scores. Both control and intervention groups demonstrated an improvement in MCQ score between pre-training and post-training testing. The mean improvement in MCQ scores was 2.6 (SD ± 3.1) in the control group (10.8%) and 3.2 (SD ± 2.3) in the intervention group (13.3%), but the difference between groups in the extent of improvement (mean 0.6; 2.5%) was not statistically significant, *t* = 1.15, *p* = 0.254 (Table 1).

**Table 1:** Test scores

	Control group: mean score (±SD; range)	Intervention group: mean score (±SD; range)	Mean difference, test statistic and *p* value
Pre-training MCQ score/24	15.0 (± 2.26; 10–20)	15.3 (± 2.05; 10–20)	0.3, *t* = − 0.79, *p* = 0.432
Post-training MCQ score/24	17.6 (± 2.93; 9–22)	18.5 (± 2.14; 13–22)	0.9, *t* = − 1.89, *p* = 0.059
Change in MCQ score/24	2.6 (± 3.14; − 5–9)	3.2 (± 2.29; − 2–8)	0.6, *t* = 1.15, *p* = 0.254
Post-training clinical test score/21	14.3 (± 2.42; 9–18)	14.2 (± 1.97; 11–20)	− 0.1, *t* = 0.130, *p* = 0.897

Average MCQ scores pre- and post-training for control and intervention groups (total points possible, 24) and post training clinical assessment scores (total points possible, 21)

The mean clinical test score for both the intervention and control groups was 14 out of a maximum possible score of 21. An independent samples *t* test revealed no significant difference between control and intervention groups in terms of clinical case scores, mean difference 0.1 (0.5%) in favour of the control group, *t* = 0.13, *p* = 0.897 (Table 1).

### Regressions of MCQ test score change on demographic variables

There was no relationship found between age, gender, number of years as a CHW, number of children in the household, number of children in the household under five and MCQ score change in either group (see Additional file 6).

We did however find a statistically significant positive correlation (Pearson’s *r* = 0.26, *p* = 0.03) linking years of education to improvement in test scores in the control group, which was not present in the intervention group. When included in a multiple regression including data for all CHWs and intervention group as a dummy variable, years of education was positively associated with MCQ change scores, with a beta coefficient of 0.33 (*p* = 0.02). The interaction effect between intervention group and MCQ change scores was not statistically significant, but the beta coefficient of − 0.32 (*p* = 0.10) implies that the effect of years of education on MCQ change scores is concentrated in the control group. The full results of the regression are shown in the additional file (see Additional file 6).

Pearson’s correlation coefficient between pre-training MCQ scores and MCQ change scores was − 0.46 (*p* < 0.001), suggesting that CHWs with the lowest pre-training scores improved the most. This would most likely be expected as these CHWs had the greatest “headroom” for improvement.

### CHW feedback and experiences

Ninety-four percent (*n* = 59) of CHWs from the intervention group responded to the feedback questionnaire. Ninety-eight percent (*n* = 58) of CHWs who responded to the open question, “What was your overall experience in using the tablet as a training tool?”, stated that it was a positive experience with quotes such as “It made me happy” and “It was good” demonstrating this.

In response to the question, “Did you have any issues using the tablet?”, 11 CHWs answer “Yes”. Battery life of the tablets was the most commonly cited problem (*n* = 6), followed by difficulties in pausing the videos (*n* = 3).

Two CHWs stated they had difficulty using the tablets, with one stating, “I kept quiet even though I did not know how to use it”. In both cases, the CHWs who were unable to use the tablets stated that other CHWs helped them to resolve the issues they had.

## Discussion

Previous studies have suggested mHealth tools are a potential means to deliver training to health workers in resource-poor countries [12, 26]. Given the lack of rigorous quantitative studies evaluating mHealth as an on-going training tool in sub-Saharan Africa, our pilot study adds to this body of literature, highlighting the feasibility and potential of a tablet-based refresher training program for CHWs in rural Uganda.

### Feasibility of delivering iCCM training via Android tablets

One of the major challenges facing CHW programs is the maintenance of skills and knowledge [7, 8, 27]. The traditional approach to training involves CHWs attending a 5-day in-person iCCM training course often held in a central location. Once the initial 5-day training course is complete, they can sometimes wait several months to receive a 1-day refresher-training course. The ability to provide regular refresher training depends on the resources of the organisation responsible for training and maintaining CHWs, such as the number of trained staff available to deliver training. The tablet-based program described in our study is one way to potentially address some of the issues surrounding the provision of refresher training. Tablet-based training requires fewer supervisors to deliver training and is less time consuming when compared to traditional methods, since all training material is hosted on the tablets. Delivering training through instructional videos also helps to ensure training material is delivered in a consistent manner, using up-to-date iCCM guidelines and helps to ensure the quality of training remains consistent. Training videos can be checked for accuracy by key stakeholders, such as representatives from the MoH, to ensure they accurately reflect local policies and guidelines.

The tablet-based method of training also offers the potential for CHWs to access refresher training in a flexible manner at a time and place that suits them, with the open-ended questionnaire feedback revealing positive feedback from the majority of CHWs assigned to the intervention group. This is important, since CHWs in Uganda are volunteers and to attend training days involves leaving their jobs. The flexibility offered by mHealth training programs has already been highlighted in the literature as means of more efficient use of time, therefore increasing opportunities for health workers in LMICs to engage in other income-generating activities [28]. The flexibility of tablet-based training may be particularly beneficial to marginalised groups, such as women, who have to balance household duties with attending training. This warrants further exploration in in-depth qualitative work using participatory action research methodologies.

### Design and development of mHealth tools for training CHWs

From a design standpoint, by working with CHWs from an early stage, we were able to deliver culturally appropriate training material. Engaging key stakeholders at every level, from CHWs through to the MoH, is vitally important to ensure the needs of learners are met and to avoid duplication of efforts. A review by Aggarwal et al. highlighted the importance of engaging CHWs in the process of design and development of mHealth tools to ensure the user interface is intuitive and easy to understand [29]. It is also important that mHealth programs take into consideration the age, level of education and previous technology use of those for whom the program is targeted at [30, 31]. We also ensured that the videos were produced in the local language, featuring a local physician. Previous studies investigating the use of mobile technologies have suggested that creating content in the local language helps contribute to the sense of ownership and understanding [13].

Although there was no significant difference between the control and intervention groups in terms of pre- and post-training MCQ test score or post-training clinical assessment scores, in the group receiving traditional training there was some evidence of a positive relationship between years of education and improvement in MCQ test scores. This relationship was not present in the group receiving tablet-based training. This might suggest that the traditional style of didactic classroom-based teaching is more suited to CHWs who have more experience of the formal education system and are more familiar with this type of learning environment and style of teaching. It might therefore be useful to adopt a blended style learning program, i.e., supplementing traditional style training with tablet-assisted training for those CHWs with fewer years of formal education. The very nature of mHealth training methods means difficult concepts can be reviewed simply by rewinding a segment of video, as well as enabling CHWs to cover the material at their own pace which is not possible in the classroom environment.

By using videos as the means of delivering training, we were able to focus on concepts that are traditionally poorly taught through didactic or text-based teaching. Previous studies have demonstrated that the referral of sick children from the community in Mukono is poor [32]. This is due to multifaceted reasons; however, one that is commonly cited in the literature is the lack of awareness of the importance of making a referral to a health centre or hospital [33]. This was a point that was emphasised during the video training modules. Finally, although videos are sometimes used in traditional training by using a projector, frequent power outages often mean that this cannot be reliably used. By hosting videos on the tablets, relying on mains electricity does not become a limiting factor, since each tablet is charged using solar chargers.

### Technical difficulties and considerations

Although the Omni Med staff hosted a half-day digital literacy course on how to use the tablets, two CHWs stated that they had difficulties using the tablets and did not know how to work them. Interestingly, they also stated that other CHWs helped them to use the tablets, perhaps suggesting that the community bond in CHW networks was one factor in helping to address technical issues. Of the nine CHWs who experienced difficulties with the tablets, the majority of issues surrounded technical factors, such as poor battery life (*n* = 6) and inability to pause videos (*n* = 3). This highlights the challenges of balancing hardware quality with costs in a resource-limited setting.

### Strengths and limitations

This is one of the first studies to assess the use of mHealth as an education tool for CHWs in Uganda. In addition, it helps to highlight the important issues and challenges surrounding the provision of refresher training to CHWs, which is an important issue not just in Uganda, but also in LMICs more generally [34].

In terms of study design, we randomised at a cluster level, but analysed at a CHW level due to the low number of clusters: we deemed this approach sufficient for a pilot trial, but appreciate that we may have missed a clustering effect. Several of the study’s limitations surround the use of assessment tools. Firstly, since the assessment tools were designed by our research team, they are unvalidated and can therefore be only accepted at face value. Secondly, it should also be noted that the clinical assessment tool was only carried out as a post-training assessment. This was due to local resource limitations and financial costs. We are therefore unable to comment on change in score between pre- and post-testing for this component of the assessment. Finally, given that the same test was used to assess knowledge before and after training, thus there is a limitation of reactivity bias where CHWs may have learnt from the test, not from the training, or were potentially “primed” on certain aspects to focus on during the training. We tried to minimise the latter risk by not informing CHWs that they would receive the same test post-training. These issues help to highlight the wider issue regarding the best way to measure the impact of training programmes. We used a pragmatic measure to assess knowledge and skills; however, in future studies it would perhaps be worth recording markers such as the number of correctly referred cases of pneumonia to health facilities as a way of determining change in clinical practice or evaluating the use of mHealth programmes through a more interpretivist lens, especially in “politicised situations where goals and success criteria are contested” [35].

Although we sought the views of participants in an informal manner throughout the design and development process and explored their experiences through the use of open-ended questionnaires, research by Winters et al. calls for better attempts at capturing “users’ experiences of systems” and attempts at measuring how mHealth tools position CHWs within their society [36]. Future studies should therefore attempt to explore and measure the socio-cultural impacts of mHealth-facilitated training on CHWs and the communities they serve.

A further limitation of the study is that we did not collect data on the total number of times each video was viewed by individual CHWs. It would have potentially been interesting to correlate such data with test scores. Other researchers may be interested in using software, such as OppiaMobile developed by Digital Campus, which allows program creators to host CHW training material on an open access mobile platform. Once an internet connection becomes available, tracking and quiz scores from training materials hosted on the platform are sent back to the server for trainers to track the progress of their learners [37].

We must also caveat that although the CHWs across all three sub-counties were generally representative of volunteer CHWs in Uganda, there are likely to be some individual variations in CHW programmes, given that multiple different NGOs take responsibility for the provision of training. The government of Uganda have recognised this as an issue and are currently in the process of implementing a centralised model of CHW training to try and control for quality assurance [38].

Finally, the duration for which the CHWs received the tablets was only 5 days. Many of the CHWs commented on how they would have preferred the tablets for a longer period of time. We are therefore unable to comment on long-term knowledge retention from this current study, as well as long-term engagement with the training platform.

## Conclusion

Although there was no significant difference between intervention and control groups in terms of improvement in assessment scores on both the theoretical and clinical case testing, we found that providing refresher training via low-cost Android tablets to CHWs in rural Uganda was feasible and well received.

With Africa’s expanding cadre of CHWs, we need to better understand how to design, deliver and evaluate effective on-going training programs. Future studies should consider how training programs might empower CHWs as agents for behaviour change, and how this is best measured is also important to consider how pilot programmes utilising mHealth, such as the one we have described, transition to scale and large-scale evaluations are particularly important in this respect.

By adopting an interdisciplinary approach through collaborative work between educationalists, global health practitioners and government agencies, we are more likely to make progress towards addressing these important issues and help empower CHWs in their increasingly important roles.

## Additional files

Additional file 1: Consort diagram. (PDF 87 kb)

Additional file 2: Pre-and post-test knowledge MCQ. (PDF 67 kb)

Additional file 3: Post training clinical scenarios. (PDF 94 kb)

Additional file 4: Evaluation questionnaire. (PDF 33 kb)

Additional file 5: Demographic data. (PDF 57 kb)

Additional file 6: Univariate analysis and regression analysis. (PDF 107 kb)

## Abbreviations

CHW: Community health worker
iCCM: Integrated Community Case Management
LMIC: Low- and middle-income country
MCQ: Multiple choice questions
MoH: Ministry of Health
USD: United Stated Dollars
