# Supplementary figures and images for: The use of low-cost Android tablets to train community health workers in Mukono, Uganda, in the recognition, treatment and prevention of pneumonia in children under five: a pilot randomised controlled trial
Source: Hum Resour Health. 2018 Sep 19;16:49. doi: 10.1186/s12960-018-0315-7 (PMC6146528; doi:10.1186/s12960-018-0315-7)

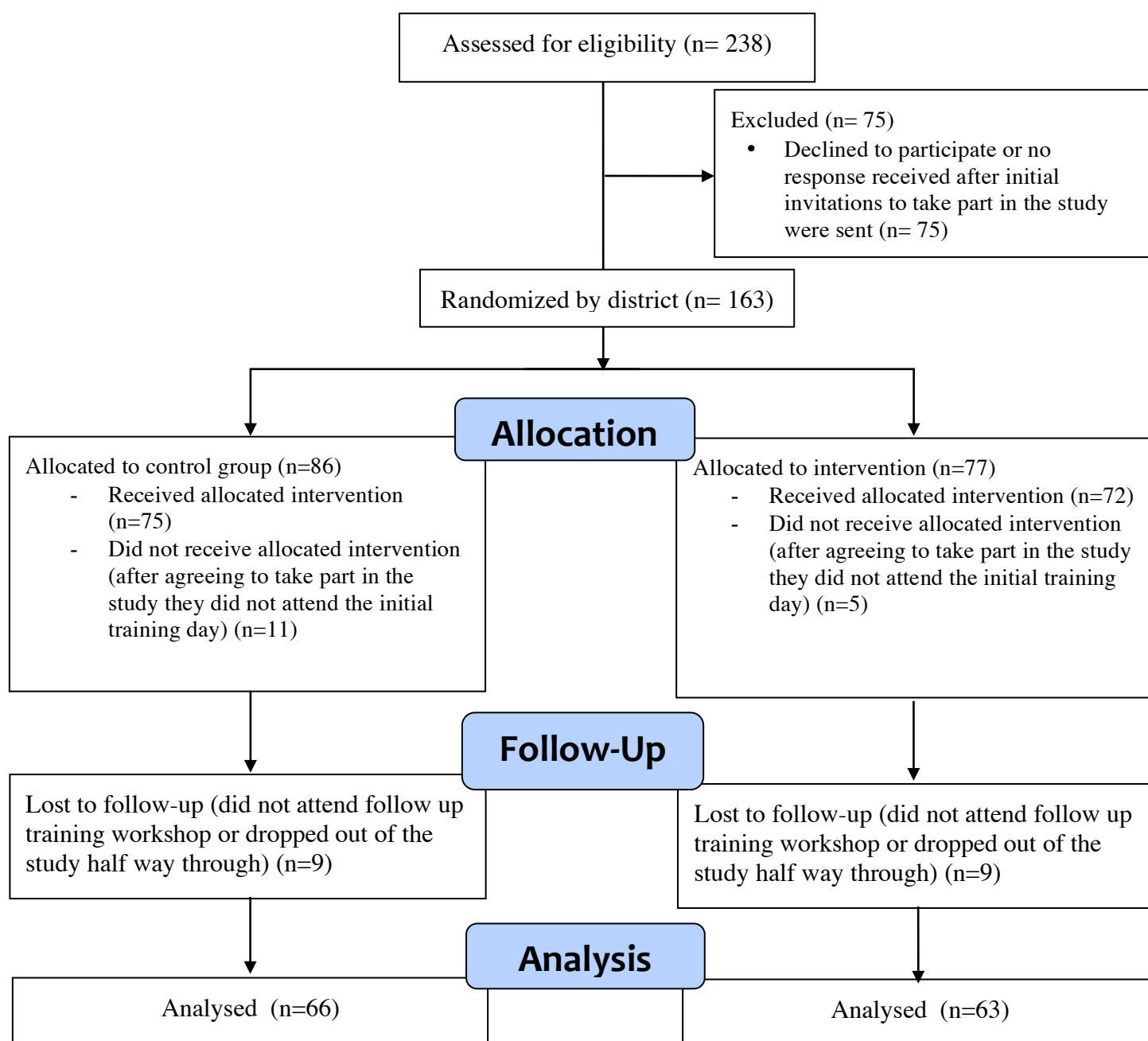

Supplement: Supplementary file 1 — Consort diagram. (PDF 87 kb) [file 12960_2018_315_MOESM1_ESM.pdf]
