# Supplementary material for: The use of low-cost Android tablets to train community health workers in Mukono, Uganda, in the recognition, treatment and prevention of pneumonia in children under five: a pilot randomised controlled trial
Source: Hum Resour Health. 2018 Sep 19;16:49. doi: 10.1186/s12960-018-0315-7 (PMC6146528; doi:10.1186/s12960-018-0315-7)
Supplement: Supplementary file 2 — Pre-and post-test knowledge MCQ. (PDF 67 kb) [file 12960_2018_315_MOESM2_ESM.pdf]

Date____
VHT Study Number (Identifier)______________________________________
Parish____
Subcounty____

REMEMBER TO USE YOUR SICK CHILD JOB AID IF YOU NEED TO. ANSWER ALL QUESTIONS.

Circle the correct answer from the following questions:

1) Pneumonia is an infection of the lungs?

   True

   False

2) Pneumonia could cause a cause a child to die.

   True

   False

3) Pneumonia is most commonly caused by viruses?

   True

   False

4) What are the 3 most important symptoms that might indicate a child might have pneumonia?

   a. Cough, fast breathing, headache
   b. Cough, fast breathing, chest in-drawing
   c. Fast breathing, abdominal pain, fever
   d. Abdominal pain, fever, no appetite

5) If a child is crying and restless you should wait until they are calm before assessing their breathing rate.

   True

   False

6) You should be watching the timer whilst you count the child's breathing rate.

   True

   False

7) If a child has a high temperature and is shaking, they might have pneumonia even if they are not breathing fast.

True

False

8) For each scenario state whether the rate of breathing is fast or normal. (Circle the correct answer)

a) A 6 month old with breathing rate of 40 breaths per minute. Is the child's breathing rate normal or fast?

Fast

Normal

b) A 5-day old with breathing rate of 80 breaths per minute. Is the child's breathing rate normal or fast?

Fast

Normal

c) Age 3 months, Rate 55 and 60 (60 is the rate recorded on the second measurement) Is the child's breathing rate normal or fast?

Fast

Normal

d) A 4-year-old with breathing rate of 32 breaths per minute. Is the child's breathing rate normal or fast?

Fast

Normal

e) Age 10 months. Rate 75 and 70 (70 is the rate recorded on the second measurement)

Fast

Normal

f) Age 2 years Rate 50 and 56 (56 is the rate recorded on the second measurement)

Fast

Normal

9.According to iCCM guidelines which antibiotic should be used to treat a child with pneumonia?

a. Coartem
b. Zinc
c. Amoxicillin
d. Cotrimoxazole

10.The following can help prevent a child from getting pneumonia? Select true or false as your answer:

a.) Limit the child's exposure to smoke and pollution by cooking outside

True

False

b.) Encourage the mother to breastfeed her child

True

False

c.) Visit the witch doctor with the child

True

False

d.) Keep the child up to date with immunizations

True

False

e.) Wash hands properly with soap and water before handling a baby or child

True

False

11. You should give a child who has HIV a tablet called cotrimoxazole to prevent them getting pneumonia?

True

False

12.What is the correct Amoxicillin dosage for 8 months old child with pneumonia?

a. Green pack: 2 tablets (500mg total), twice a day for 5 days
b. Red pack: 2 tablets (500mg total), twice a day for 10 days
c. Green pack: 1 tablet (250 mg), twice a day for 5 days
d. Red pack: 1 tablet (250mg), twice a day for 5 days

13.What is the correct Amoxicillin dosage for 3-year old child with pneumonia?

a. Green pack: 2 tablets (500mg total), twice a day for 5 days
b. Red pack: 2 tablets (500mg total), twice a day for 3 days
c. Green pack: 1 tablet (250 mg), twice a day for 5 days
d. Red pack; 1 tablet (250mg), twice a day for 5 days

14.What else should be taught to the mother at your home visit? (Select one answer)

a. The child must finish all of the antibiotics as instructed, even if the child's symptoms are improving
b. Tell her not to feed her child
c. Tell her to put her child to bed to help slow his breathing
d. Tell her to stop the antibiotics as soon as the child looks better

15.If you cannot remember the doses of antibiotic what is the best way to help you find out?

a. Guess the dose
b. Do not give antibiotics at all
c. Refer to the Sick Child Job Aid
d. Ask someone in your village
