# Supplementary material for: The use of low-cost Android tablets to train community health workers in Mukono, Uganda, in the recognition, treatment and prevention of pneumonia in children under five: a pilot randomised controlled trial
Source: Hum Resour Health. 2018 Sep 19;16:49. doi: 10.1186/s12960-018-0315-7 (PMC6146528; doi:10.1186/s12960-018-0315-7)
Supplement: Supplementary file 3 — Post training clinical scenarios. (PDF 94 kb) [file 12960_2018_315_MOESM3_ESM.pdf]

Date____
VHT Study Number (Identifier)____
Parish____
Subcounty____

REMEMBER TO USE YOUR SICK CHILD JOB AID IF YOU NEED TO. ANSWER ALL QUESTIONS.

Case 1:

Ben is age 7 months. He has been coughing for 2 weeks. He does not have a fever or blood in his spit.

Watch the video and count the number of breaths he takes in one minute to work out if he has fast breathing or not. Use the SCJA if you needed to.

1) How many breaths did Ben take in one minute?

2) Does Ben have fast breathing? *(Circle one answer)*

   Yes No

3) Does Ben have chest in-drawing? *(Circle one answer)*

   Yes No

4) Does Ben need treatment in the community, a referral to the health center, or will his illness resolve itself? *(Circle one answer)*

a. Treat in Community by VHT
b. Refer to Health Centre
c. His illness will resolve and he does not need any follow up

Case 2:

Lord is 7 months old. He has a cough and fever.

His breathing rate is much slower. You determine that he has pneumonia but can be treated in the community with antibiotics.

1) Which antibiotic would you use, or advise the family to purchase? (*Circle one answer)*

   a. Amoxicillin
   b. Cotrimoxizole
   c. Ciprofloaxcin

2) What dose of antibiotic would you need to give him or tell them to give him? *(Circle one answer)*

a. Red Pack - 250mg, twice daily for 5 days
b. Green Pack – 500mg, twice a day, for 5 days
c. Purple Pack – 1000mg, three times a day, for 10 days

3) If you will not be starting the antibiotics yourself, when will you tell the family to start treatment with antibiotics? *(Circle one answer)*

a. Immediately, within 24 hours
b. Next week
c. Tell them not to start antibiotics

Case 3:

Josh is age 6 months. He has been coughing for 2 days.

He has no blood in his spit, no fever and no chest in-drawing.

Watch the video and count the number of breaths he takes in one minute to work out if he has fast breathing or not. Use the SCJA if you need to.

1) How many breaths did Josh take in one minute?

2) Does Josh have fast breathing*? (Circle one answer)*

Yes No

3) Does Josh need treatment in the community, a referral to the health centre, or will his illness resolve itself? (*Circle one answer)*

a. Treat in Community by VHT

b. Refer to Health Centre

c. His illness will resolve and he does not need any follow up

Case 4:

Mugisa is 6 months old. He has a cough and fever, but no fast breathing.

He has NO chest in-drawing.

You determine that he has pneumonia but does not need to be referred to a health center; you will treat him in the community.

1) Which antibiotic would you need to treat him with? *(Circle one answer)*

a. Amoxicillin

b. Cotrimoxizole
c. Ciprofloaxcin

2) What dose of the above antibiotic would you or his family need to give him? *(Circle one answer)*

a. Red Pack - 250mg, twice daily for 5 days
b. Green Pack – 500mg, twice a day, for 5 days
c. Purple Pack – 1000mg, three times a day, for 10 days

Case 5:

Pooh is age 4 years. She has been coughing for 2 weeks. She has had NO blood in her spit. After watching her chest, you determined that he does NOT have chest in-drawing. You have examined her and determined she DOES have a fever.

Watch the video and count the number of breaths she takes in one minute to work out if she has fast breathing or not. Use the SCJA if you needed to.

1) How many breaths did Pooh take in one minute?

2) Does Pooh have fast breathing? *(Circle one answer)*

Yes or No

3) Does Pooh's case need management in the community by a VHT, a referral to the health center, or will her illness resolve itself*? (Circle one answer)*

a. Management in Community
b. Refer to Health Center
c. Illness Will Resolve

Case 6:

James is the same age as Pooh, 4 years old. He has had a cough and fever for one week. He has no blood in his spit and his respiratory rate is not fast.
He should be treated in the community by you the VHT.

1) Which antibiotic would you need to treat him with or advise his family to purchase for him? (*Circle one answer)*

a. Amoxicillin
b. Cotrimoxizole
c. Ciprofloaxcin

2) What dose of the above antibiotic should he receive? (*Circle one answer)*

a. Red Pack - 250mg, twice daily for 5 days
b. Green Pack – 500mg, twice a day, for 5 days
c. No antibiotic needed

Case 7:

Jenny is age 11 months. She has been coughing for 3 days, but has had no blood in her spit. You have examined her and determined she does have a fever.

After watching her chest, you determined that she does NOT have chest in-drawing.

Watch the video and count the number of breaths she takes in one minute to work out if she has fast breathing or not. Use the SCJA if you need to.

1) How many breaths did Jenny take in one minute?

2) Does Jenny have fast breathing? (Circle one answer)

Yes or No

3) How should Jenny be managed? (Circle one answer)

   a. Manage in Community by VHT
   b. Refer to Health Center
   c. Do not do anything

4) If Jenny can be managed in the community by the VHT, how should she be managed initially? *(Circle one answer)*

   a. Treat with antibiotics
   b. No treatment with antibiotics now, but follow-up check in 2 days
   c. Do nothing at all
