# Supplementary material for: The use of low-cost Android tablets to train community health workers in Mukono, Uganda, in the recognition, treatment and prevention of pneumonia in children under five: a pilot randomised controlled trial
Source: Hum Resour Health. 2018 Sep 19;16:49. doi: 10.1186/s12960-018-0315-7 (PMC6146528; doi:10.1186/s12960-018-0315-7)
Supplement: Supplementary file 4 — Evaluation questionnaire. (PDF 33 kb) [file 12960_2018_315_MOESM4_ESM.pdf]

Post-training questionnaire for intervention group CHWs

1. What were your experiences of using the tablet?

2. Did you have any difficulties using the tablet?

3. If you had difficulties using the tablet what were they?

4. Did any one help you use the tablet?

5. If you liked using the tablet, what did you like about it?
