# Supplementary material for: The use of low-cost Android tablets to train community health workers in Mukono, Uganda, in the recognition, treatment and prevention of pneumonia in children under five: a pilot randomised controlled trial
Source: Hum Resour Health. 2018 Sep 19;16:49. doi: 10.1186/s12960-018-0315-7 (PMC6146528; doi:10.1186/s12960-018-0315-7)
Supplement: Supplementary file 5 — Demographic data. (PDF 57 kb) [file 12960_2018_315_MOESM5_ESM.pdf]

Additional file 5. Demographic data.

	**Control Group** (n=66) (*Mean;±S.D; Range*)	**Intervention Group** (n=63) (*Mean;±S.D; Range*)	**P-value**
Gender (Female:Male)	38:28	33:30	0.678
Age	44.6 (±12.5; 48)	43.7 (±10.3; 50)	0.658
Number of Years as a CHW	5.9 (±1.66; 8)	5.6 (±1.16; 7)	0.250
Number of Years of Education	9.2 (±2.5; 10)	8.7 (±2.52; 12)	0.382
Average number of children in their household	5.3 (±2.63; 14)	5.1 (±3.72; 21)	0.820
Average number of children in their household age under five	1.6 (±1.17; 5)	1.4 (±1.4; 7)	0.327

Legend. A breakdown of demographic data between the control and intervention groups.
