# Supplementary material for: The use of low-cost Android tablets to train community health workers in Mukono, Uganda, in the recognition, treatment and prevention of pneumonia in children under five: a pilot randomised controlled trial
Source: Hum Resour Health. 2018 Sep 19;16:49. doi: 10.1186/s12960-018-0315-7 (PMC6146528; doi:10.1186/s12960-018-0315-7)
Supplement: Supplementary file 6 — Univariate analysis and regression analysis. (PDF 107 kb) [file 12960_2018_315_MOESM6_ESM.pdf]

Additional file 6. Univariate analysis and regression analysis.

Univariate analysis (dependent variable change in MCQ scores):

Demographic	Method of analysis	Control group (N=66)	Intervention group (N=63)
Age	Pearson's correlation coefficient	R=-0.12, p=0.347	R=-0.09, p=0.471
Gender	Independent samples t-test	T=1.44, p=0.155	T=1.30, p=0.198
Years as a CHW	Kendall's tau-b	Tau-b=0.01, p=0.883	Tau-b=-0.02, p=0.826
Number of children<5	Kendall's tau-b	Tau-b=-0.01, p=0.963	Tau-b=0.01, p=0.906
Years of education	Pearson's correlation coefficient	R=0.02, p=0.91	R=0.26, p=0.033

Regression analysis (dependent variable change in MCQ scores):

Explanatory variable	Coefficient	p-value	95% Confidence interval
Intervention group	3.45	0.056	-0.08 to 6.98
Years of education	0.33	0.016	0.06 to 0.59
Interaction	-0.32	0.103	-0.69 to 0.06
Constant	-0.40	0.752	-2.93 to 2,12

R-squared = 0.055, F=2.44, p=0.067

Legend. An outline of the univariate and regression analysis of MCQ test score changes on demographic variables.
